# Observed Agreement Problems between Sub-Scales and Summary Components of the SF-36 Version 2 - An Alternative Scoring Method Can Correct the Problem

**DOI:** 10.1371/journal.pone.0061191

**Published:** 2013-04-12

**Authors:** Graeme Tucker, Robert Adams, David Wilson

**Affiliations:** 1 SA Department of Health, Adelaide, South Australia, Australia; 2 Discipline of Medicine, University of Adelaide, Adelaide, South Australia, Australia; 3 The Queen Elizabeth Hospital, Woodville, South Australia, Australia; Research and Development Corporation, United States of America

## Abstract

**Purpose:**

A number of previous studies have shown inconsistencies between sub-scale scores and component summary scores using traditional scoring methods of the SF-36 version 1. This study addresses the issue in Version 2 and asks if the previous problems of disagreement between the eight SF-36 Version 1 sub-scale scores and the Physical and Mental Component Summary persist in version 2. A second study objective is to review the recommended scoring methods for the creation of factor scoring weights and the effect on producing summary scale scores

**Methods:**

The 2004 South Australian Health Omnibus Survey dataset was used for the production of coefficients. There were 3,014 observations with full data for the SF-36. Data were analysed in LISREL V8.71. Confirmatory factor analysis models were fit to the data producing diagonally weighted least squares estimates. Scoring coefficients were validated on an independent dataset, the 2008 South Australian Health Omnibus Survey.

**Results:**

Problems of agreement were observed with the recommended orthogonal scoring methods which were corrected using confirmatory factor analysis.

**Conclusions:**

Confirmatory factor analysis is the preferred method to analyse SF-36 data, allowing for the correlation between physical and mental health.

## Introduction

The SF-36 and the shorter form SF-12 health status questionnaires have been used extensively in international studies to obtain summary measures of health status. The origin of the instruments has an extensive and well-founded methodological history deriving from the Medical Outcomes Study conducted by the RAND Corporation [1]. However, international concern has been raised questioning the validity of the recommended orthogonal scoring methods of Version 1 of the SF-36 to produce Physical and Mental Component Summary scores (PCS & MCS) [2]-[9]. However, these scoring methods remain in widespread use, indeed they are the default scoring approach around the world. Given the instruments subscales and summary scores are used by national agencies to guide policy [10] and medical authorities to guide treatment and intervention decisions, [11], it is important that questions of validity are addressed to achieve best investment decisions. The creation of Version 2 of the instrument led to a number of refinements to question item response categories, layout and norming of the questionnaire. Data items for the role physical and role emotional items, which contribute substantially to PCS and MCS summary scores were expanded from dichotomous yes/no responses to five point Likert scales. New norms were derived from the 1998 US population, which have since been updated to 2009. [12]. No substantial changes were made to the recommended scoring methods [12], so the question remains as to whether or not the commercial Version 2 still produces summary scores that are at variance with the underlying sub-scale scores [5]. The major putative problem with the recommended scoring methods is they do not allow for a correlation between physical and mental health in creating the summary scores; an issue that is not consistent with the health literature. Epidemiological and clinical studies have shown a strong connection between physical and mental health [13]–[18]. People with depression often have worse physical health, as well as worse perception of their health [16], a characteristic that would affect their reporting of self-related health. Tucker et al [5], acknowledged this connection in the SF-36 version 1 by demonstrating that the use of the recommended orthogonal scoring methods, which do not allow for the correlation, created important discrepancies between the PCS and MCS and their underlying sub-scale scores, and that this could be corrected by use of confirmatory factor analysis (CFA). Given the extensive use of Version 2 [12] it is important to again compare recommended orthogonal scoring methods with CFA, assess if the problems found in Version 1 persist and resolve which methods may best analyse Version 2 to produce summary scores consistent with the sub-scales.

A second important question relating to the use of the SF-36 is whether or not cross-country comparisons of health status are valid using the recommended United States (US) factor scoring coefficients in the development of the PCS and MCS. The developers of the SF-36 Version 2 advocate use of US factor score weights in creating the PCS and MCS in other countries [19]. This has the effect of artificially inflating or deflating these components for local decision making, which could confuse investment decisions in health for other countries. Given the potential differences of health status, the distribution of health and the perception of health in different countries, the question arises as to whether or not PCS and MCS scores should be based on country specific weights and, therefore, be free to vary from country to country, in order to accurately reflect the sub scale scores generated. Using US factor score coefficients standardises scores of each country to the US sub-scale score profile [20], which is possibly different to the sub-scale score profile of the country conducting the study. The important question to be answered is whether or not comparisons across countries are best made on the basis of country specific weighting coefficients?

Our aim was to assess whether previous problems of disagreement between the eight SF-36 Version 1 sub-scale scores and the Physical and Mental Component Summary scales (PCS and MCS) persist in version 2 of the instrument. A second study objective is to review the recommended scoring methods for the creation of factor scoring weights and the effect on producing summary scale scores

## Methods

### Statistical background and methodological issues

In producing the SF-36 component summaries (PCS and MCS) from the SF-36 data there are two main options for rotation of factors. This is done depending on whether or not the investigator believes the factors to be correlated (oblique) or uncorrelated (orthogonal). The recommended scoring methods for the SF-36 are based on orthogonal rotations, but we will argue that this creates data agreement problems and that there is strong support for adopting an oblique approach.

The items of the SF-36 are set out in Table 1.

**Table 1:** Detailed items of the SF-36 version 2.

Sub-scale	Item	Short description	Question
Physical	a3a	Vigorous activities	The following questions are about activities that you might do
Functioning	a3b	Moderate activities	during a typical day. As I read each item, please tell me if your
	a3c	Lift/Carry groceries	health now limits you a lot, limits you a little, or does not limit you
	a3d	Climb several flights	at all, in these activities.
	a3e	Climb one flight	1 = Yes, limited a lot
	a3f	Bend, Kneel	2 = Yes, limited a little
	a3g	Walk kilometre	3 = No, no limited at all
	a3h	Walk half a kilometre	
	a3i	Walk 100 metres	
	a3j	Bathe, Dress	
Role	a4a	Cut down time	The following four questions ask you about your physical health
Physical	a4b	Accomplished less	and your daily activities. During the past four weeks, how much
	a4c	Limited in kind	of the time have you.?
	a4d	Had difficulty	1 = All of the time
			2 = Most of the time
			3 = Some of the time
			4 = A little of the time
			5 = None of the time
Bodily Pain	a7	Pain-magnitude	How much bodily pain have you had during the past four weeks?
			1 = None
			2 = Very mild
			3 = Mild
			4 = Moderate
			5 = Severe
			6 = Very severe)
	a8	Pain-interfere	During the past four weeks, how much did pain interfere with your
			normal work, including both work outside the home and
			housework?
			1 = Not at all
			2 = Slightly
			3 = Moderately
			4 = Quite a bit
			5 = Extremely
General	a1	EVGFP rating	These first questions are about your health now and your current
Health			daily activities. Please try to answer every question as accurately
			as you can. In general, would you say your health is:
			1 = Excellent
			2 = Very good
			3 = Good
			4 = Fair
			5 = Poor
	a11a	Sick easier	Now I'm going to read you a list of statements. After each one,
	a11b	As healthy	please tell me if its definitely true, mostly true, mostly false, or
	a11c	Health to get worse	definitely false. If you don't know just tell me.
	a11d	Health excellent	1 = Definitely true
			2 = Mostly true
			3 = Don’t know
			4 = Mostly false
			5 = Definitely false
Vitality	a9a	Full of life	The following questions are about how you feel and how things
	a9e	Energy	have been with you in the past four weeks. As I read each
	a9g	Worn out	statement, please give me the one answer that comes closest to the
	a9i	Tired	way you have been feeling. Would you say all of the time, most of
			the time, some of the time, a little of the time or none of the time?
			1 = All of the time
			2 = Most of the time
			3 = Some of the time
			4 = A little of the time
			5 = None of the time
Social	a6	Social-extent	During the past four weeks, to what extent has your physical health
Functioning			or emotional problems interfered with your normal social activities
			with family, friends, neighbours or groups? Has it interfered:
			1 = Not at all
			2 = Slightly
			3 = Moderately
			4 = Quite a bit
			5 = Extremely
	a10	Social-time	During the past four weeks, how much of the time has your
			physical health and emotional problems interfered with your social
			activities like visiting friends and relatives? Would you say:
			1 = All of the time
			2 = Most of the time
			3 = Some of the time
			4 = A little of the time
			5 = None of the time
Role	a5a	Cut down time	The following three questions ask about your emotions and your
Emotional	a5b	Accomplished less	daily activities. During the past four weeks, how much of the time
	a5c	Not careful	have you.?
			1 = All of the time
			2 = Most of the time
			3 = Some of the time
			4 = A little of the time
			5 = None of the time
Mental	a9b	Nervous	The following questions are about how you feel and how things
Health	a9c	Down in dumps	have been with you in the past four weeks. As I read each
	a9d	Calm	statement, please give me the one answer that comes closest to the
	a9f	Felt down	way you have been feeling. Would you say all of the time,
	a9h	Happy	most of the time, some of the time, a little of the time or none of the time?
			1 = All of the time
			2 = Most of the time
			3 = Some of the time
			4 = A little of the time
			5 = None of the time

**Figure 1:**
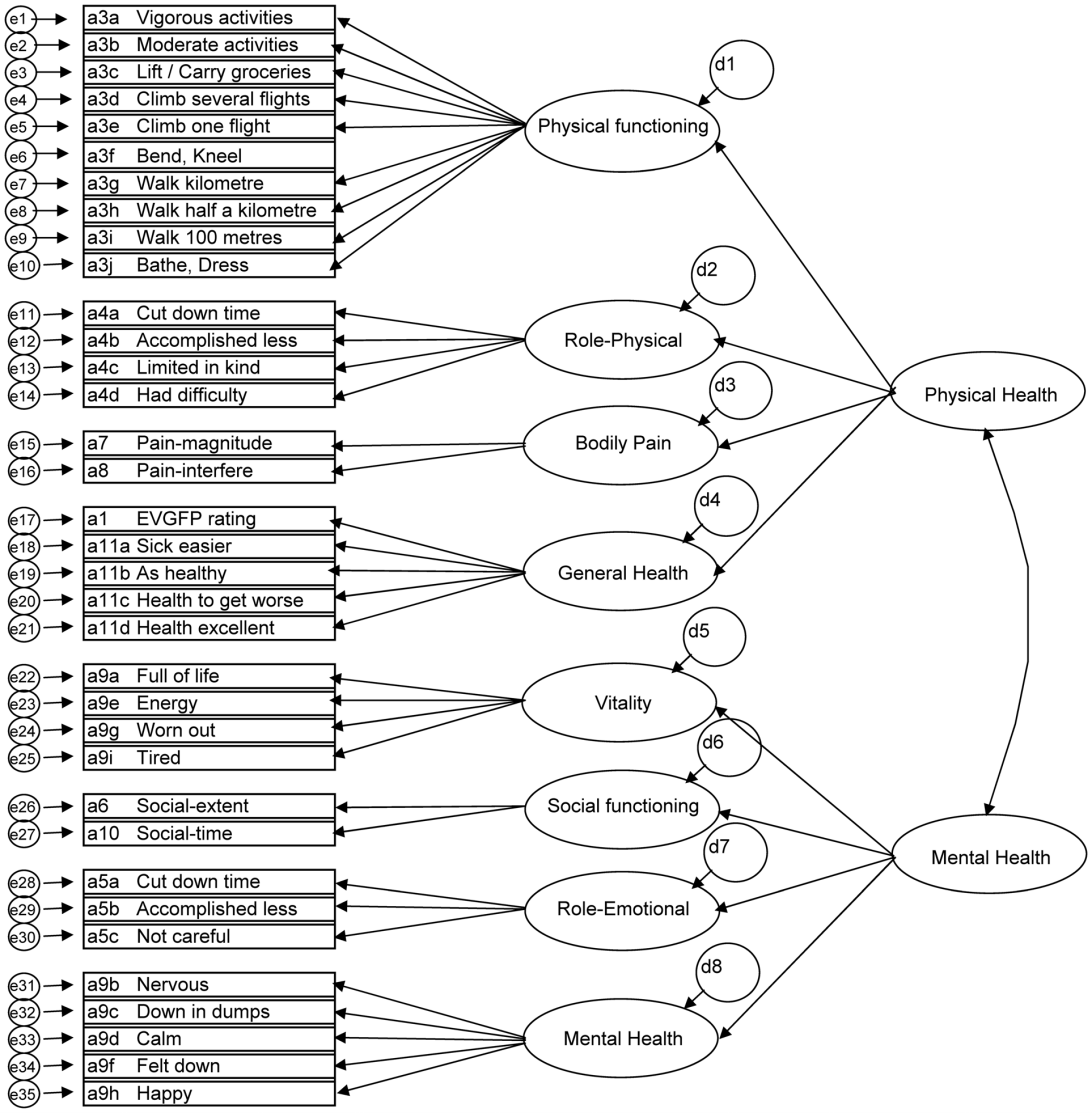
Hypothesised structure of SF-36 Health Dimensions and the Summary Physical (PCS) and Mental (MCS) Health Measures.

A hypothetical factor structure has already been documented for the SF-36 [21]. This formed the basis of the model we evaluated, except that we allowed physical and mental health to be correlated (see Figure 1). It was therefore possible to fit a second order confirmatory factor analysis (CFA). The model fit was the full measurement model, using items re-coded as detailed in the SF36 scoring manual [20], with the exception that integer values of the items were retained so that they could be modeled using polychoric and tetrachoric correlations in LISREL V8.7. The above model was fit on 3,014 observations with no missing data for any items. The data produced using the CFA was compared with an analysis using the recommended orthogonal scoring methods [22].

Exploratory factor analysis (EFA) based on z-scores of the sub-scales, employing a principal components (PCA) extraction and an orthogonal rotation of factors was used by the developers to produce the SF-36 scoring coefficients for the component summary scores. This model cannot be directly fit using CFA software as the model is unidentified. However, using MacDonald's “echelon form” [23] where one non-significant path is constrained to zero, fit measures for the EFA model were generated in Stata [24]. It should be pointed out that the EFA model uses Pearson correlations of z-scored normally distributed data for the eight sub-scale scores, whereas the CFA model uses polychoric correlations of the 35 data items involved in the calculation of the SF-36 scores. Also the Akaike Information Criteria (AIC) value from the CFA model fit in LISREL V8.7 [25] is based on the Satorra-Bentler Chi-squared value, and the AIC from the EFA model fit in Stata SE V12 [24] is based on the model chi-square which is -2*log likelihood. To produce a fair comparison of the two models, the AIC was re-calculated for the CFA model based on the value of -2*log likelihood.

Hawthorne et. Al [22]. have published population norms for the transformed subscale scores from the 2004 SA Health Omnibus Survey [26], and they used the traditional scoring approach of Ware et al to produce factor score weights for the calculation of the Australian SF-36 summary scores. We also used these published norms and weights to produce subscale and summary PCS and MCS scales, distributed N(50,100), based on the traditional orthogonal method, for comparison with the CFA, using the 2008 SA Health Omnibus Survey data set.

Given the complexity of decisions made in the process of the CFA analysis the following methodological explanations are provided.

First, Rigdon & Ferguson [27] have shown that Maximum Likelihood (ML) estimation based on a polychoric correlation matrix is insufficient to correct for the problems associated the type of data in this study. For this reason weighted least squares (WLS) estimation is preferred. Further, Mindrilla [28] concluded that Diagonally Weighted Least Squares (DWLS) is superior to ML for the analysis of ordinal data.

Nye & Drasgow [29] consider that WLS and DWLS are both from the Asymptotically Distribution Free (ADF) family of estimators, and require similar large size samples. They investigated sample sizes from 400 to 1600. Flora & Curran contradict this paper, concluding that DWLS (they call it robust WLS) is superior to WLS in almost all situations, especially when the model is complex or the sample is small (n = 100). The largest sample size they considered was 1000 [30].

Forero et. al [31] compared unweighted least squares (ULS) and diagonally weighted least squares (DWLS) as alternatives to WLS for estimating Confirmatory Factor Analysis (CFA) models with ordinal indicators in a Monte Carlo study, and concluded that ULS was preferable, but if this did not converge then DWLS should be used, even in small samples (they examined sample sizes of 200. 500, and 2000). WLS was eliminated from consideration due to the requirement for very large sample sizes.

For our analysis, we have a moderate sample size of 3014. We attempted to use ULS as recommended by Forero et al [31], but this did not converge for the SF-36 model. We therefore chose to use DWLS to fit the model for SF-36. The model for SF-12 converged using ULS.

For maximum likelihood estimation of multivariate normal data, fit measure cutoffs have been set out by Hu and Bentler [32] as: Root Mean Square Error of Approximation (RMSEA) < = 0.06, Standardised Root Mean Square Residual (SRMSR) < = 0.08, Tucker Lewis Index (TLI) > = 0.95, Comparative Fit Index (CFI) > = 0.95. TLI is also known as the Non-Normed Fit Index (NNFI).

Nye & Drasgow [29] concluded that the fit measures and cutoffs in use for ML estimation of multivariate normal data do not apply to ADF estimators. They based their proposals for interpretation of fit measures on DWLS estimators of dichotomous indicators in CFA via tetrachoric correlations. They used Monte Carlo computer simulation to study the effects of model misspecification, sample size, and non-normality on fit indices generated from DWLS estimation on dichotomous data. The study consisted of a 3 (model misspecification)×3 (degree of nonnormality)×3 (sample size) design. This is based on simulations of sample sizes of 400, 800, and 1600, using values of 0, 0.5, and 1.75 for skewness, and 0, 1.0, and 3.75 for kurtosis.

The reader is indirectly invited to extend the results to ordinal data and polychoric correlations, but this is an assumption. They have set out how to calculate cutoffs for fit measures for different situations (i.e. different levels of skewness, kurtosis, sample size, and required type I error rates). They only considered positive skewness in their calculations. They found that CFI & TLI were almost always near 1, and did not provide any discrimination regarding the fit of these models. Therefore, they recommend judging fit for these models based on their calculated cutoffs for RMSEA and SRMSR.

Flora & Curran [30] found that “there were few to no differences found in any empirical results as a function of two category versus five category ordinal distributions.” This conclusion supports the generalisation of Nye & Drasgow's work from tetrachoric to polychoric correlations. They also found that DLWS produced more accurate estimates of the model chi-square, and therefore all of the fit measures that are based on it. In WLS estimation, the “inflation of the test statistic increases Type I error rates for the chi-square goodness-of-fit test, thereby causing researchers to reject correctly specified models more often than expected.”. In this sense, Flora and Curran argue the opposite of Nye & Drasgow, [29] who proffer the advice that goodness-of-fit criteria need to be tightened up to avoid accepting inadequate models.

Nye and Drasgow [29] considered sample sizes up to 1600, and the formulae they provide produce complex roots when applied to our dataset, despite our skewness and kurtosis parameters lying within the ranges used in their simulations. We consider that this is because our sample size is much greater than the experience of their simulations.

Since the Nye and Drasgow [29] formulae fail to provide real valued cutoffs in our dataset, and Flora and Curran [30] argue for less stringent rather than more stringent fit criteria, we are comfortable using the maximum likelihood criteria advanced by Hu and Bentler [32] to assess model fit in this analysis, with the exception that Nye and Drasgow's advice regarding the non-discrimination of the TLI and CFI fit indices is accepted. We have therefore based our acceptance of the model on an RMSEA< = 0.06 and a SRMSR< = 0.08.

### Statistical analysis

The 2004 South Australian Health Omnibus Survey dataset was used as the basis for the production of scoring coefficients [26]. This is the earliest Australian population survey available which included version 2 of the SF-36 health status questionnaire. In this representative population survey n = 3,014 adults aged 15 years or older were interviewed, all of whom provided full information for the SF-36. This is the same dataset as used by Hawthorne et. al. [22]. The data items were recoded as per the instructions of the SF-36 scoring manual [20].

The confirmatory factor analyses were fit on polychoric correlations in LISREL V8.7 [25] software. The model for SF-36 is a second order confirmatory factor analysis model. Unfortunately LISREL does not produce factor score weights for second order factors. The AMOS package [33] does produce these coefficients, but does not model polychoric correlations. Therefore we applied the AMOS formula for the generation of factor score weights to the outputs provided by LISREL to calculate factor score weights for version 2 of the SF-36. The AMOS formula is given by W = B S^−1^ where W is the matrix of factor score weights, S is the fitted variance covariance matrix of the observed variables in the model, and B is the matrix of covariances between the observed and unobserved variables [33]. As pointed out by Joreskog [34] latent variable scores should be independent of the estimation method used to fit the model. The use of this formula satisfies this requirement.

The existence of factor score weights for all of the 35 items in the calculation of the summary scores based on the model is explained by the fact that all variables have an effect on both physical and mental health by virtue of the correlation between them, which is allowed for in the model.

**Figure 2:**
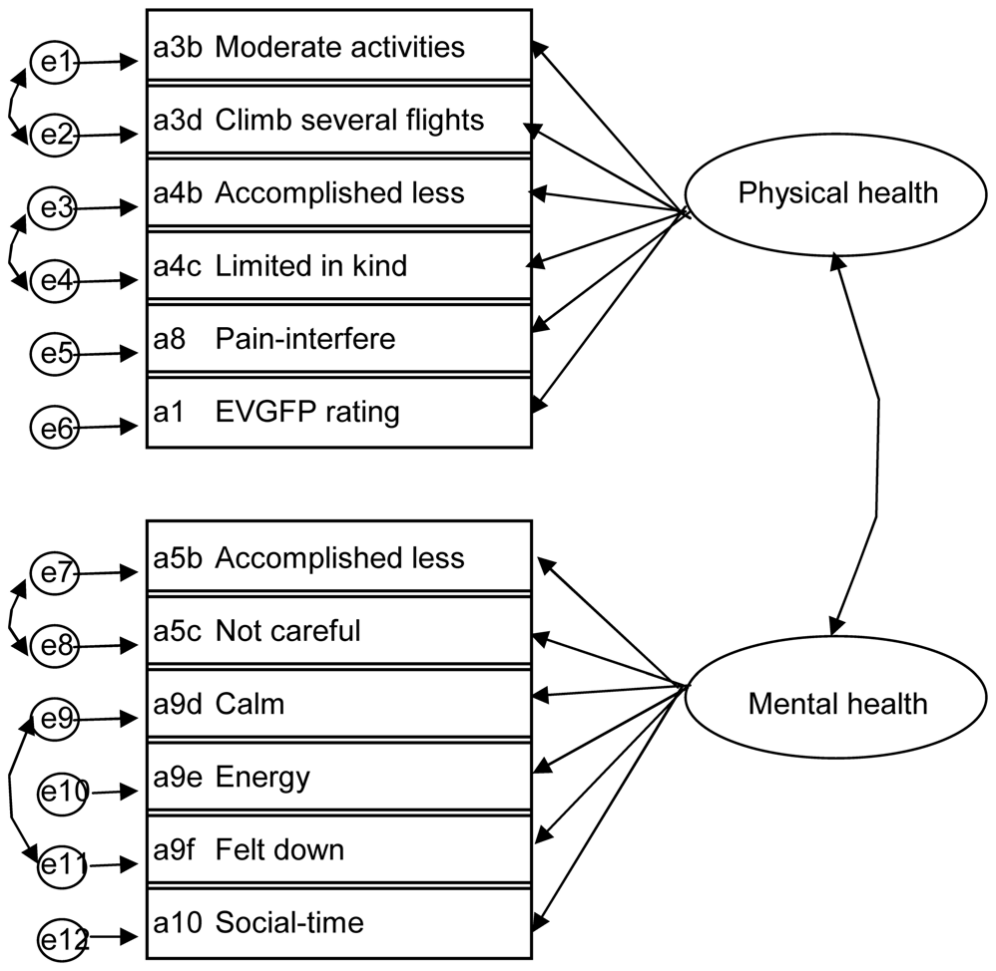
Hypothesised structure of SF-12 Summary Physical (PCS) and Mental (MCS) Health Measures.

A similar approach was used to model the SF-12 variables (see Figure 2). Models were again fit to produce the factor score weights in a confirmatory factor analysis. The data were recoded as per the instructions of the SF-36 scoring manual [20], with the exception that question eight of the SF-36 was recoded according to the instructions where question seven is not answered. This is because question seven is not asked in collecting the SF-12 data items. This resulted in 3,014 records being available to the analysis. In the model, correlations were allowed among the error terms for items from the same SF-36 sub-scale, because items from the same sub-scale, could reasonably be expected to be more closely correlated with each other than with the other items of the SF-12.

Comparisons of the PCS and MCS mean scores were based on agreement with the underlying subscales for both the orthogonal rotation and CFA. It was postulated that any sub-group summary score that was higher or lower than average should be in statistical agreement with the underlying subscales that contribute to that summary score. For comparison we used four age groups (<30 years, 30–49 years, 50–69 years and 70+ years) and four medication groups (no medication, physical health medication, mental health medication and both physical and mental health medication). Both sets of scores were based on the 2008 SA Health Omnibus Survey data. Since all scores were hypothesised to be distributed normally with a mean of 50 and a standard deviation of 10, comparisons were made assuming equal variances. Mean scores for four age groups and four medication groups were compared with the complementary groups to determine which age and medication groups had scores which were higher or lower than average scores. Similar comparisons were also made for the eight sub-scale scores. For each age and medication group comparisons of summary scores were made with the underlying sub-scale scores using independent groups t-tests. These analyses were carried out using SPSS Version 19 [35].

## Results

The traditional orthogonal EFA model had an RMSEA = 0.104, SRMSR = 0.022, CFI = 0.972, TLI = 0.940, and AIC = 58497.72. This can be compared with our CFA model with RMSEA = 0.049, SRMSR = 0.053, CFI = 0.995, TLI = 0.9908, and AIC = 50495.37. From these fit measures it can be seen that the CFA model provides a much superior fit to the data than the EFA model with an orthogonal rotation. We bear in mind the view of Nye and Drasgow [29] that the CFI and TLI are constrained to be near unity in the analysis of polychoric correlations for ordinal data.

Table 3.5 of SF-36 Physical and Mental Health Summary Scales: A User's Manual [21] provides the Pearson product-moment correlations of the sub-scales for the general US population. This table provides sufficient information to test the fit of the original orthogonal EFA model employed by the developers of the scale. Using the same methods as above, the orthogonal EFA of the original US data had an RMSEA = 0.092, SRMSR = 0.028, CFI = 0.971, TLI = 0.938, and AIC = 47130.90. The original US model therefore shows a similar degreee of lack of fit as the same model fit to Australian data by Hawthorne [22].

The coefficients generated by the CFA analysis for the SF-36 are set out in Table 2. The model had a Chi-square of 53511.3 on 551 degrees of freedom, the size of which is explained by the large sample size. The Satorra-Bentler [36] scaled chi-square was 4648.5. The model had an RMSEA of.050 (90% confidence interval.048 to.051), a probability of close fit of 0.6522, and a standardised root mean square residual of 0.076. The Non-Normed Fit Index was 0.9904 and the Comparative Fit Index was 0.9911. The estimate of the correlation between physical and mental health was 0.73 (p<0.001).

**Table 2:** Australian weighting coefficients for the SF-36 version 2.

	PCS	MCS
A3A	0.0258	0.0025
A3B	0.0623	0.0120
A3C	0.0445	0.0025
A3D	0.0680	0.0070
A3E	0.2366	0.0263
A3F	0.0268	0.0031
A3G	0.1044	0.0087
A3H	1.0457	0.1367
A3I	0.1675	0.0262
A3J	0.0169	0.0021
A4A	0.5621	0.0697
A4B	1.2488	0.1658
A4C	1.9280	0.2391
A4D	2.2187	0.2757
A7	0.2566	0.0352
A8	0.9124	0.1121
A1	0.4297	0.0523
A11A	0.1698	0.0212
A11B	0.2881	0.0368
A11C	0.0895	0.0119
A11D	1.2084	0.1553
A9A	0.1653	1.6617
A9E	0.1882	1.8847
A9G	0.0817	0.8083
A9I	0.0839	0.8228
A6	0.1478	1.4785
A10	0.2389	2.4014
A5A	0.1022	1.0694
A5B	0.1017	1.0106
A5C	0.0393	0.3615
A9B	0.0125	0.1300
A9C	0.0503	0.4939
A9D	0.0263	0.2478
A9F	0.0601	0.5955
A9H	0.0288	0.2983
Constant term	−0.1097	−9.6528

Based on these weights the theoretical range of the SF-36 version 2 PCS is (12.3279,59.6503), and the observed range was (13.5313,59.6503). For the SF-36 version 2 MCS the theoretical range is (5.0138,63.3733), and the observed range was (5.5778,63.3733).

The coefficients generated by the CFA analysis for the SF-12 are set out in Table 3. The model had a Chi-square of 2646.6 on 49 degrees of freedom. The Satorra-Bentler scaled chi-square was 588.4. The model had an RMSEA of 0.060 (90% confidence interval 0.056 to 0.065), a probability of close fit of 0.000, and a standardised root mean square residual of 0.075. The Non-Normed Fit Index was 0.9874 and the Comparative Fit Index was 0.9906. The estimate of the correlation between physical and mental health was 0.71 (p<0.001).

**Table 3:** Australian weighting coefficients for the SF-12 version 2.

	PCS	MCS
A1	1.3019	0.2044
A3B	1.2625	0.1984
A3D	0.6006	0.0943
A4B	3.0028	0.4730
A4C	2.9809	0.4693
A8	2.0033	0.3157
A5B	0.1863	1.8531
A5C	0.0953	0.9532
A9D	0.0800	0.7996
A9E	0.2422	2.4132
A9F	0.1370	1.3584
A10	0.4964	4.9376
Constant term	0.3833	−9.0891

Based on these weights the theoretical range of the SF-12 version 2 PCS is (12.7725,58.6031), and the observed range was (12.7725,58.6031). For the SF-36 version 2 MCS the theoretical range is (4.9811,60.6765), and the observed range was (4.9811,60.6765).

In comparing the effect of orthogonal rotation methods with confirmatory factor analysis we compared the summary scale scores with their underlying sub-scale scores for different age groups in Table 4 and for medication groups in Table 5. From the tables clear discrepancies are apparent between the traditional summary scores and their sub-scales, which are not evident using scoring coefficients derived from confirmatory factor analysis.

**Table 4:** Comparison of subscale scores and summary scores using different scoring methods, by age groups.

	<30	30–49	50–69	70+	Total
n	515	991	939	472	2917
Physical function scale -	54.7	52.7	47.5	39.9	50.0
Aust normed T-score					
Role physical scale - Aust	52.2	50.9	47.2	43.6	49.2
normed T-score					
Bodily pain scale - Aust	52.5	49.0	45.7	45.5	48.5
normed T-score					
General health scale - Aust	51.6	50.4	47.7	46.1	49.3
normed T-score					
Vitality scale - Aust	51.3	49.1	48.9	47.6	49.4
normed T-score					
Social function scale - Aust	50.4	49.3	48.4	48.3	49.2
normed T-score					
Role emotion scale - Aust	49.7	48.6	48.7	48.7	48.9
normed T-score					
Mental health scale - Aust	49.1	48.8	49.0	50.0	49.1
normed T-score					
SF-36–PCS scored using	54.1	51.6	46.6	41.5	49.5
Aust weighted T-score					
SF-36 MCS- scored using	48.3	47.9	49.6	52.0	49.0
Aust weighted T-score					
SF-36 PCS - scored using	52.6	50.8	47.2	43.9	49.3
SEM coefficients					
SF-36 MCS - scored using	51.1	49.3	48.2	47.0	49.1
SEM coefficients					
SF-12 PCS - scored using	52.6	50.8	47.0	43.6	49.2
SEM coefficients					
SF-12 MCS - scored using	51.2	49.5	48.2	47.0	49.2
SEM coefficients					

**Table 5:** Comparison of subscale scores and summary scores using different scoring methods, by medication status.

	No medication	Physical only	Mental only	Both	Total
n	1549	1120	95	153	2917
Physical function scale -	53.5	45.6	48.7	41.0	50.0
Aust normed T-score					
Role physical scale - Aust	52.2	45.8	45.9	40.6	49.2
normed T-score					
Bodily pain scale - Aust	51.6	44.9	43.7	38.7	48.5
normed T-score					
General health scale - Aust	52.4	45.8	44.1	40.6	49.3
normed T-score					
Vitality scale - Aust	51.4	47.9	41.9	40.7	49.4
normed T-score					
Social function scale - Aust	51.1	47.8	41.2	40.7	49.2
normed T-score					
Role emotion scale - Aust	50.7	48.5	37.4	38.2	48.9
normed T-score					
Mental health scale - Aust	50.3	49.2	39.0	40.1	49.1
normed T-score					
SF-36–PCS scored using	53.1	44.6	48.5	40.9	49.5
Aust weighted T-score					
SF-36 MCS- scored using	49.8	50.0	37.0	40.3	49.0
Aust weighted T-score					
SF-36 PCS - scored using	52.7	45.6	44.4	39.3	49.3
SEM coefficients					
SF-36 MCS - scored using	51.6	47.4	39.2	37.9	49.1
SEM coefficients					
SF-12 PCS - scored using	52.5	45.6	44.6	39.1	49.2
SEM coefficients					
SF-12 MCS - scored using	51.7	47.5	39.4	38.0	49.2
SEM coefficients					

Table 4 shows several discrepancies between the summary component scores and their underlying sub-scale scores when scored using orthogonal methods, as set out by Hawthorne [22]. The score for the SF-36 mental health sub-scale for those aged under thirty years is not significantly different to the overall sub-scale average (p = 0.918),. The remaining three sub-scale scores that comprise the SF-36 mental component are all significantly higher than average (role emotional (p = 0.026), vitality (p<0.001), social functioning (p = 0.005)), as are the mental component summary scores (MCS) from CFA coefficients for both SF-36 (p<0.001) and SF-12 (p<0.001), yet the MCS score, based on the original orthogonal scoring algorithm, is significantly lower than average (p = 0.035).

For those aged 30–49 years, none of the mental health sub-scales are significantly different to average (vitality (p = 0.272), social functioning (p = 0.650), role emotional (p = 0.295), and mental health (p = 0.264)), yet the MCS was significantly lower than average (p<0.001) using orthogonal scoring, but there was no significant difference for the SF-36 MCS score using CFA coefficients (p = 0.561) or SF-12 using CFA coefficients (p = 0.294).

For those aged 50–69 years, three of the mental health scales were not significantly different to average (vitality (p = 0.120), role emotional (p = 0.466), and mental health (p = 0.795)) and social functioning was significantly lower than average (p = 0.012), yet the MCS was significantly higher than average (p = 0.044) using orthogonal scoring but significantly lower than average for both SF-36 (p = 0.003) and SF-12 (p = 0.001) using CFA coefficients.

For those aged 70 years or more, the vitality scale was significantly lower than average (p<0.001), whilst the social functioning (p = 0.083), role emotional (p = 0.711), and mental health score (0.069) were not significantly different to average. The MCS scores from CFA coefficients for both SF-36 (p<0.001) and SF-12 (p<0.001) were significantly lower than average, yet the MCS score based on the original orthogonal scoring method was significantly higher than average (p<0.001). There were no inconsistencies evident by age for physical health summary scores when compared to their subscales.

Similar discrepancies arise in comparison of the component summary scores with their underlying sub-scale scores for those taking medications for either or both physical and mental health conditions. Table 5 shows that for those not taking medications no inconsistencies between sub-scales and summary scores were evident. For those taking medications for physical ailments the vitality (p<0.001) and social functioning (p<0.001) sub-scales scores were both significantly lower than average, while the role emotional score (p = 0.155) and the mental health score (p = 0.789) were not significantly different to average. This is consistent with the mental health summary scores (MCS) from CFA coefficients which were significantly lower than average for both SF-36 and SF-12 (p<0.001), yet the MCS score based on the original orthogonal scoring method was significantly higher than average (p<0.001).

Similarly, three of the physical health subscale scores are significantly lower than average for those taking medications for mental health reasons (role physical (p = 0.002), bodily pain (p<0.001), and general health (p<0.001)), while the physical functioning scale is not significantly different to average (p = 0.196). This is consistent with the physical health summary scores (PCS) from CFA coefficients which are significantly lower than average for both SF-36 (p<0.001) and SF-12 (p<0.001), yet the PCS score based on the original scoring coefficients is not significantly different to average (p = 0.380) for PCS calculated using orthogonal methods.

There were no inconsistencies evident for those taking medication for both physical and mental health problems for physical or mental health summary scores when compared to their subscales.

In summary, the CFA produced a superior fit to the SF-36 data, provided acceptable fit measures and solved agreement problems observed in the orthogonal analyses.

## Discussion

We raise two points of difference with the developers regarding the development of scoring norms and weights. First, that PCS and MCS summary scores should be based on a model that allows correlation of physical and mental health, to preserve consistency of summary scores with their underlying sub-scales. We thank an anonymous reviewer who has also pointed out that “this issue is probably more of a concern with the SF12 than the SF36. The SF36 generates subscale scores, so users can notice and evaluate the potential problems caused by orthogonally-derived summary scores. But the SF12 generates only summary scores, so the problem will be hidden from users.”. Second, that scoring norms and weights should be produced on country specific data, so that all scores are based on the same data items and have the same distributions (normal with mean 50 and standard deviation 10). This is essential for country decision making especially from summary scales for sub-groups, but further in this way all countries will produce T-scores for all sub-scales and summary scales that allow accurate international comparisons, without the need to standardise to USA factor weights

The use of US factor score weights in the calculation of summary scores seems inappropriate for other countries, because the linear combination of z-scored sub-scales using US weights results in the emphasis being placed on those sub-scales which have higher US weights. Hawthorne [22] has analysed Australian SF-36 version 2 data from the 2004 Health Omnibus Survey. His analysis replicated precisely to the methods used by the developers, but included allowances for the production of Australian norms for use in calculating the z-scores for the sub-scales, and for the calculation of Australian factor score weights from an orthogonal EFA. His analysis showed that the factor score weights produced based on Australian data were significantly different to those produced using USA data. None of the USA weights were in the 95% CI of the Australian weights. Thus the profile of locally calculated weights can be very different to the US weights and therefore the summary scores produced by locally produced weights would emphasise different sub-scales than the US weights. This results in the calculation of inaccurate summary scores when using US weights. In principal therefore, calculation of summary scores should be based on locally calculated weights. In the present study we used the Australian norms and factor score weights based on Australian data developed by Hawthorne [22] to produce the component summary scores for the traditional orthogonal scoring method. Table 2 of Hawthorne's paper also demonstrated the shortcomings of applying US norms and weights to Australian data, in that the 95% CI for all subscale T-scores and the MCS T-score excluded 50. So even if we stick to orthogonal analyses there is important and increasing evidence that strictly applying US factor score weights in the creation of summary scores is a problem for local interpretation and use of data. It is argued that the profile of locally calculated weights can be very different, as demonstrated by Hawthorne [22], and often for the valid reasons of differences in health. The aim of measuring health status should primarily be for the production of valid local scores based on country specific norms and not for the primary purpose of standardising to US data for comparison purposes. Further, if we need to compare with the US or with any other country it would best be done on the basis of subscale T-scores and summary scores based on individual data items and local population norms for the creation of factor score weights in a second order confirmatory factor analysis, so that scores are all based on the same data items and have the same distribution.

In fairness to the authors of the SF-36 they have produced a leading generic quality of life instrument and measure and there is little or no criticism about the long-term historical development of question items. The main points of contention are involved in scoring the summary scores. The question which has to be answered by other interested researchers is does the proposed CFA fix the underlying problems identified with the PCS and MCS and should US factor score weights be used for anything other than academic comparison with US data, and not for country specific estimates which may be skewed by US coefficients.

The CFA used in this analysis is based on the original data items and the orthogonal analysis on the underlying subscales. It is argued this is a reasonable comparative approach of the two methods as the data items are used to create the subscales. The main difference in the comparisons is therefore based on the methodological difference of orthogonal or oblique rotation and not on data differences. We argue the oblique rotation method is an improved way of handling the data. We further argue that the approach recommended by the developers is unsustainable in Australia, and possibly elsewhere, because the factor score weights should be free to vary from country to country in order to accurately reflect the sub-scale scores generated by the SF-36 data in each country. This point is supported by Hawthorne's analysis of the Australian data [22].

We accept that Hawthorne's findings contradict the findings of the IQOLA project [19]. Australia appears to offer divergent results to the other mainly European countries included in the IQOLA study, and we note that these analyses were conducted on different datasets. The critical point is the existence of the dataset that produced Hawthorne's results. Hawthorne's analysis satisfactorily demonstrates the need for an Australian country specific scoring algorithm. The question of the need for country specific scoring algorithtms elsewhere has not been covered by our analysis, and should be the subject of further research.

We are aware that demonstration of the inconsistencies between the sub-scales and the component summary scores in two tables (4 & 5) is not a comprehensive validation of the scoring coefficients, but we suggest there are limits to how much analysis can be squeezed into one paper.

## Conclusion

The conclusion of the study is that the problems of agreement between PCS and MCS summary scores and their underlying sub-scales identified in Version 1 of the SF-36 persist in Version 2. As identified in the Version 1 analyses [4], this occurs when a negative Z-score is multiplied by a negative coefficient, resulting in a positive score. This mathematical difficulty is compounded by the orthogonal method used, and why the authors continue to promote the method in the face of international concerns and a real world correlation between mental and physical health is not clearly understood. In a defence of the SF-36 scoring methods and the instruments accuracy, Ware and Kosinski [37], discuss the question of the PCS and MCS being rotated by orthogonal or oblique methods and ask how much physical health should be in mental health and vice versa. If, however, exploratory factor analysis using maximum likelihood extraction and oblique rotation were used, this would estimate the hypothetical factor structure and the data would determine how much mental health is contained in physical health and vice versa.

In Ware and Kosinski's [37] defence of the SF-36 they also contend “results based on summary measures should be thoroughly compared with the SF-36 profile….,” before drawing any conclusions. If we followed this advice for the above analyses of Version 2 data (and also for Version1) we would conclude the disagreement between scales and summary scores is consistent using orthogonal modeling and is based on a mathematical artefact.
